# A Review on the Synthesis and Bioactivity Aspects of Beauvericin, a *Fusarium* Mycotoxin

**DOI:** 10.3389/fphar.2018.01338

**Published:** 2018-11-20

**Authors:** Qinghua Wu, Jiri Patocka, Eugenie Nepovimova, Kamil Kuca

**Affiliations:** ^1^College of Life Science, Yangtze University, Jingzhou, China; ^2^Department of Chemistry, Faculty of Science, University of Hradec Kralove, Hradec Kralove, Czechia; ^3^Toxicology and Civil Protection, Faculty of Health and Social Studies, Institute of Radiology, University of South Bohemia České Budějovice, České Budějovice, Czechia; ^4^Biomedical Research Centre, University Hospital, Hradec Kralove, Czechia

**Keywords:** beauvericin, biosynthesis, bioactivity, anticancer, *Fusarium*

## Abstract

Beauvericin (BEA) is an emerging *Fusarium* mycotoxin that contaminates food and feeds globally. BEA biosynthesis is rapidly catalyzed by BEA synthetase through a nonribosomal, thiol-templated mechanism. This mycotoxin has cytotoxicity and is capable of increasing oxidative stress to induce cell apoptosis. Recently, large evidence further shows that this mycotoxin has a variety of biological activities and is being considered a potential candidate for medicinal and pesticide research. It is noteworthy that BEA is a potential anticancer agent since it can increase the intracellular Ca^2+^ levels and induce the cancer cell death through oxidative stress and apoptosis. BEA has exhibited effective antibacterial activities against both pathogenic Gram-positive and Gram-negative bacteria. Importantly, BEA exhibits an effective capacity to inhibit the human immunodeficiency virus type-1 integrase. Moreover, BEA can simultaneously target drug resistance and morphogenesis which provides a promising strategy to combat life-threatening fungal infections. Thus, in this review, the synthesis and the biological activities of BEA, as well as, the underlying mechanisms, are fully analyzed. The risk assessment of BEA in food and feed are also discussed. We hope this review will help to further understand the biological activities of BEA and cast some new light on drug discovery.

## Introduction

**Figure 1:**
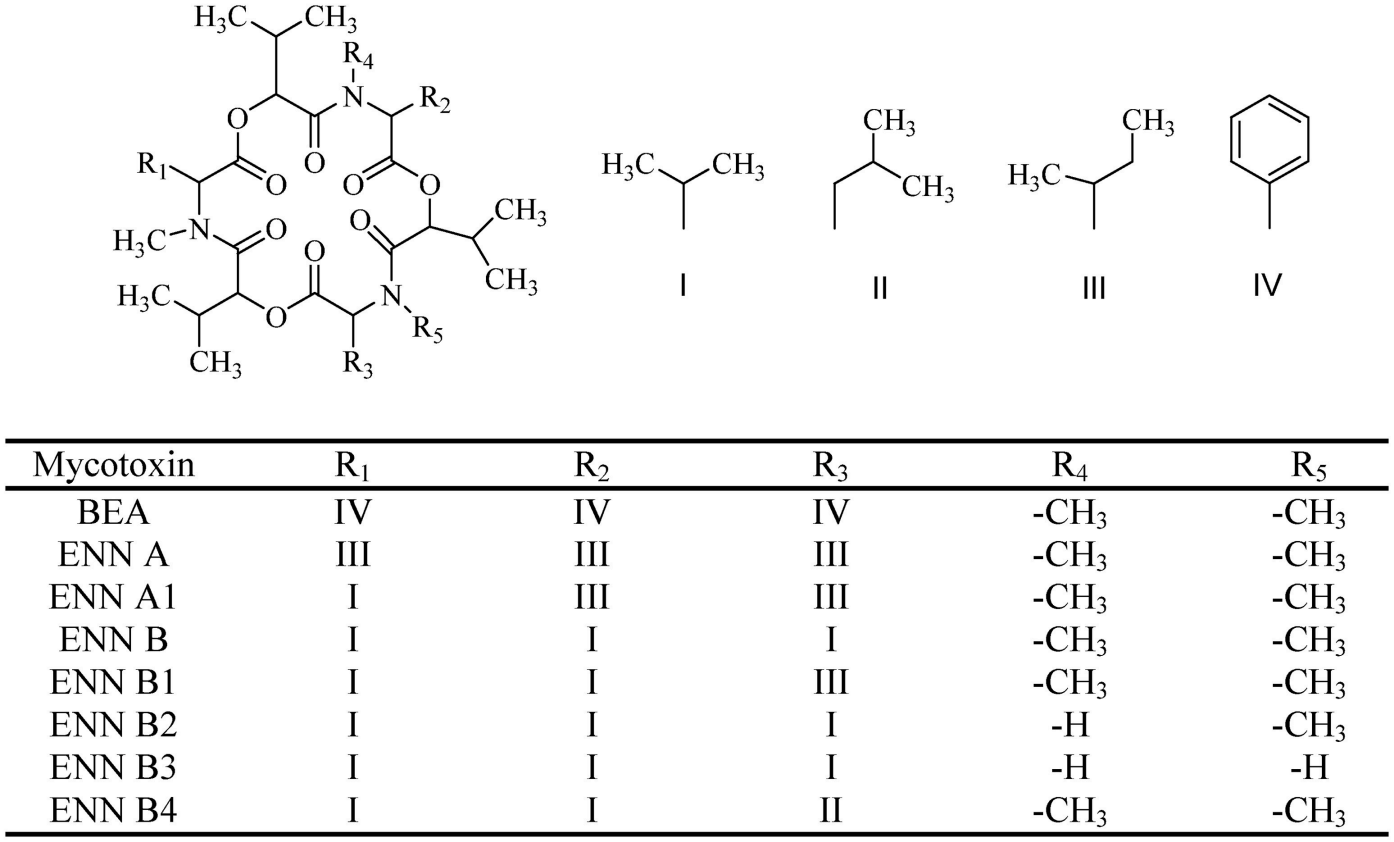
The chemical structure of beauvericin (BEA) and enniatins (ENNs).

Beauvericin (BEA) (Figure 1) is a cyclic hexadepsipeptide that is synthesized by various toxigenic fungi, including several *Fusarium* species (Wang and Xu, 2012; Tao et al., 2015; Patocka, 2016). BEA can be produced by different *Fusarium* species in different regions. For example, in the USA and South Africa, *F. circinatum* is the main BEA producing fungi, whereas, in Europe, *F. sambucinum* and *F. subglutinans* are the major ones (more details in Mallebrera et al., 2018). As a mycotoxin, BEA is an important natural contaminant in many bowls of cereal and cereal based products (Shin et al., 2009; Juan et al., 2013a,b). The contamination of BEA is a serious problem in Southern Europe (Santini et al., 2012). Notably, BEA is toxic to human tissues and cells and shows cytotoxicity at a concentration lower than that for aflatoxin B1 (Svingen et al., 2017). BEA induces the generation of reactive oxygen species (ROS) and leads to an increase of oxidative stress, which causes cell apoptosis (Ferrer et al., 2009). The channel forming ability of BEA selectively directs a flux of cations, particularly Ca^2+^, into the cells. The resulting increased intracellular Ca^2+^ levels might be, at least in part, responsible for their cytotoxicity (Lemmens-Gruber et al., 2000). BEA activates apoptosis via the internal mitochondrial pathway and influences several cellular signaling pathways and regulators including MAPK, NF-κB, and p53 (Feudjio et al., 2010; Qadri et al., 2011).

Interestingly, this mycotoxin possesses a wide variety of biological properties. It shows promising antibacterial and antifungal activities and potentiates other antifungal agents therapies (Zhang et al., 2007). Importantly, BEA can inhibit cancer cell proliferation and induce cell apoptosis via activating a Ca^2+^-mediated mechanism (Jow et al., 2004). Moreover, at sub-cytotoxic concentrations, this mycotoxin can also inhibit directional cancer cell motility (haptotaxis) (Zhan et al., 2007). Haptotaxis is important for the development of new blood vessels in tumors (angiogenesis) (Mizukami et al., 2018). The molecular mechanism(s) of the biological action of BEA is related to its ionophoric activity. BEA rapidly increases ion permeability (especially Ca^2+^) in biological membranes (from extracellular to intracellular) and acts as a cytotoxin. Up to date, BEA has been reported to cause significant cytotoxicity in a variety of cancer cell lines and oxidative stress seems to be one potential mechanism in the induction of cancer cell death at the molecular level (Tonshin et al., 2010). BEA is also genotoxic and induces apoptosis due to the activation of mitochondrial-death pathway (Celik et al., 2010). Recent studies further show that BEA is stable enough to cross the blood-brain barrier, indicating neurotoxicity (Taevernier et al., 2016a).

**Figure 2:**
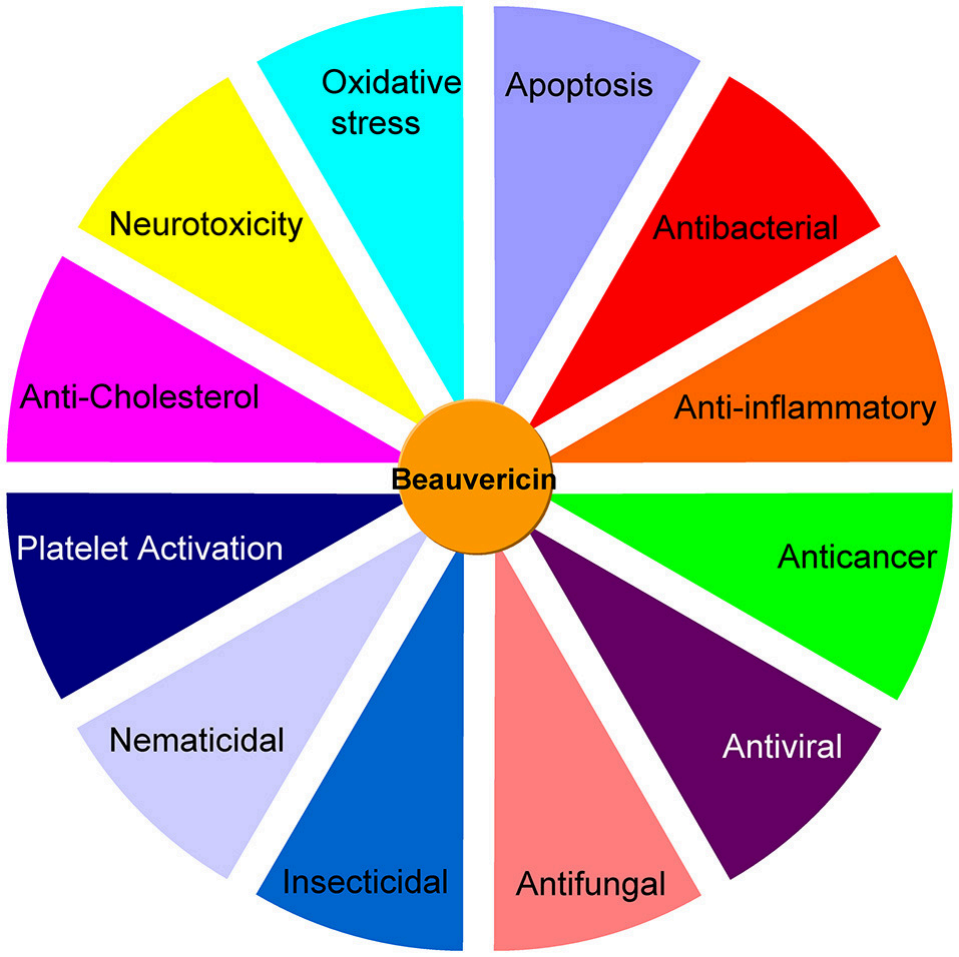
A summary of the different bioactivities of beauvericin.

The *in vitro* cytotoxicity implies that BEA can be potentially used for cancer therapeutics. This compound inhibits drug efflux pumps, is non-mutagenic and inhibits bone resorption, which suggests it as a potential drug candidate to fight disseminated cancer (Feudjio et al., 2010). Thus, as reported, BEA has many biological activities including antibacterial, antifungal, anticancer, anti-inflammatory, insecticidal, nematicidal, platelet activation, and anti-cholesterol activities (Figure 2). All these characteristics are quite crucial for the development of medicine and pesticides. However, up to date, these reports are scattered, and there is rarely a recent review article available discussing the various biological activities of BEA as well as their potential mechanisms. Thus, in this review, we have updated and discussed the biosynthesis and the major biological activities of BEA.

## Chemistry

BEA (CAS 26048-05-5. M.W. 783.957) is a cyclic hexadepsipeptide that contains three D-hydroxy-isovaleryl and three N-methyl-phenylalanyl residues in an alternating sequence (Hamill et al., 1969) (Figure 1). BEA belongs to the enniatins (ENNs) antibiotic family and it is structurally similar to the ENNs, which are also produced by *Fusarium* species. BEA differs from ENNs in the nature of the N-methylamino acid. Their bioactivities are quite different due to the difference between BEA and ENNs (Shin et al., 2009; Yoo et al., 2017) (Figure 1). The absence of any chargeable groups in cyclic hexapeptide explains the poor water solubility and the low chemical reactivity of BEA, which also is characterized by a three-fold axis of symmetry (Logrieco et al., 2002).

## Synthesis

**Figure 3:**
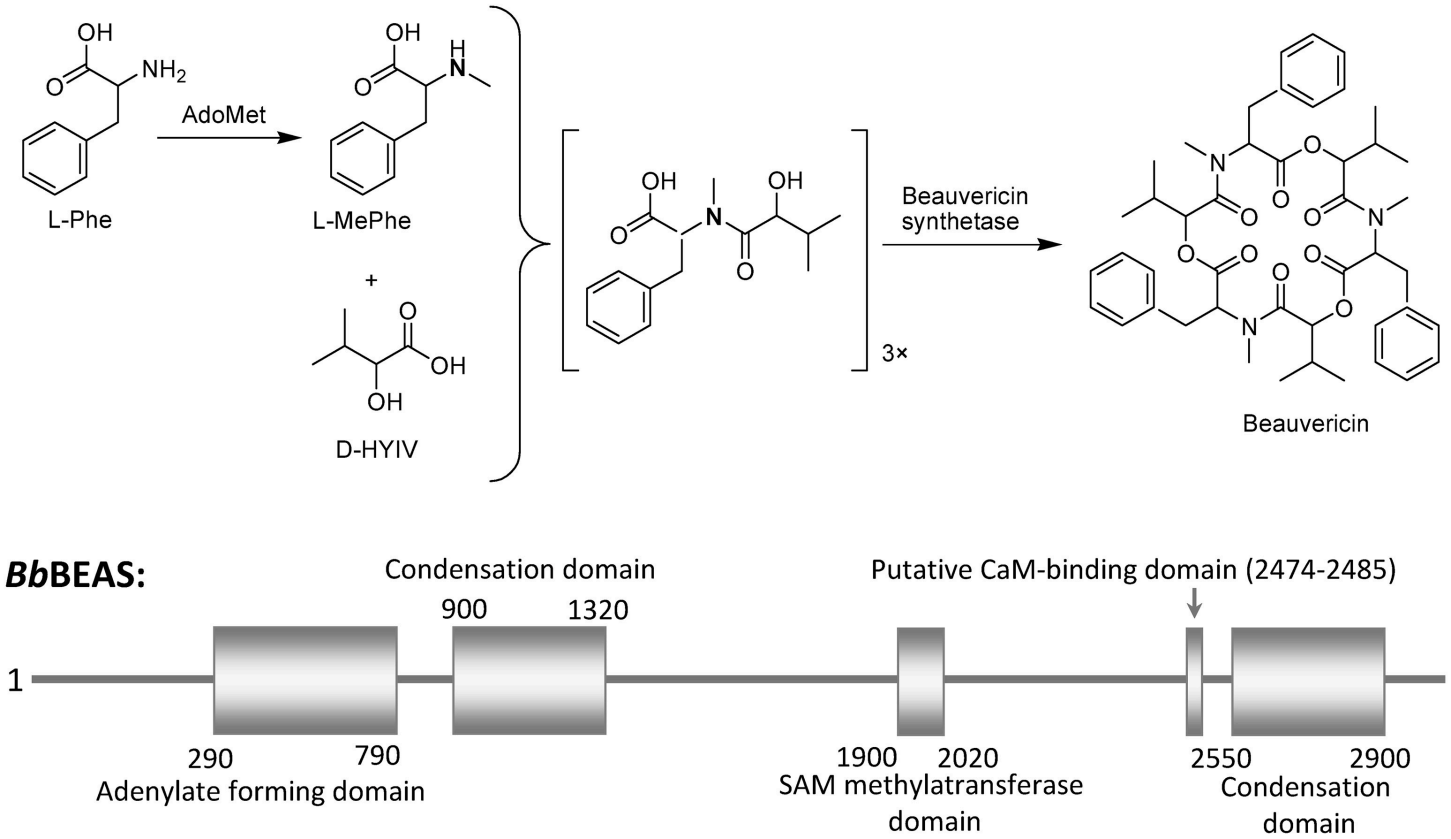
Beauvericin biosynthesis scheme.

BEA is produced by many fungi including *Beaveria bassiana* (Hamill et al., 1969; Peczynska-Czoch et al., 1991) and *Fusarium* spp. (Logrieco et al., 1998). The necessary components in EBA biosynthesis are amino acid L-phenylalanine (L-Phe), the hydroxy acid D-hydroxyisovaleric acid (D-HYIV), ATP/Mg^2+^, and S-adenosyl-methionine (AdoMet), which is the source of the methyl group for the L-phenylalanyl residues (Wang and Xu, 2012; Zobel et al., 2016). Basically, BEA biosynthesis is rapidly catalyzed by BEA synthetase (BEAS) through a nonribosomal, thiol-templated mechanism (Kopp and Marahiel, 2007; Xu et al., 2008; Steiniger et al., 2017). BEAS consists of a single polypeptide chain (molecular mass 250 kDa) and a calmodulin binding motif (Peeters et al., 1988)*. B. bassiana* BEAS (*Bb*BEAS) can interact with Ca^2+^ sensor calmodulin (CaM) in a Ca^2+^-dependent manner (Kim and Sung, 2018). *In vitro*, CaM-binding assay showed that the His-tagged *Bb*BEAS binds to CaM in a Ca^2+^-dependent manner. CaM binding to *Bb*BEAS also induces the conformational change of interacted proteins, which will further affect its enzyme activity. Therefore, *Bb*BEAS is a novel CaM-binding protein in *B. bassiana*. The schematic representation of the BEA biosynthesis is shown in Figure 3.

## Risk assessment of beauvericin in food and feed

There is a high occurrence of BEA in grains and wheat-based products like pasta, infant formulas, breakfast cereals, and biscuits, with incidences between 40 and 90% (Santini et al., 2012; Stanciu et al., 2017). In recent years, BEA has frequently been reported in different countries (South Africa, Norway, China, Croatia, Poland, Spain, Morocco…) as contaminants of, especially wheat, rye, oats, barley, and rice (Decleer et al., 2018). Seed-borne infection of wheat with *F. proliferatum* leads to contamination of wheat kernels with BEA (15–55 μg/kg) (Guo et al., 2016). In the study of Quiles et al. (2016), around 3% of refrigerated pizza dough were contaminated by BEA with level 22.39 μg/kg. BEA (1.3 ng/L) is also detected in Swiss wastewater treatment plant (Schenzel et al., 2012). Notably, BEA is a common contaminant in Danish cereals and show high hepatoxicity on a high-content imaging platform (Svingen et al., 2017). Because this fungi often contaminate food, feedstock, and animal feed, BEA is a risk factor for environmental health. Although the cereal-based food and feed contamination of Fusaria is huge, and although BEA is regularly found in these products (Hietaniemi et al., 2016; Svingen et al., 2017; Beccari et al., 2018; Carballo et al., 2018), the EFSA Panel on Contaminants in the Food Chain (CONTAM Panel, 2014) concluded that acute exposure to BEA and ENNs do not indicate concern for human health.

Basically, BEA has less occurrence in grains from cooler climates while a higher contamination level of BEA is usually reported in Southern Europe and Morocco (max 59 mg/kg in maize) (Santini et al., 2012). The BEA levels in rice from the local market of Morocco are varied between 3.8 and 26.3 mg/kg (Sifou et al., 2011; Fraeyman et al., 2017). The finding of 10.6 mg/kg of BEA in a rice-based infant cereal sample from Morocco should be highlighted (Mahnine et al., 2011). Mycotoxins contamination in wheat-based products in Romania for direct human consumption were evaluated and 32% of them presented BEA (Stanciu et al., 2017). In corn grits from the Japanese market, BEA was found in 34% of the samples. The maximum concentration of BEA was 26.1 μg/kg (Yoshinari et al., 2016). In China, BEA was detected in 96.9% of the commercial pet food with levels ranging 0.2–153.4 μg/kg (Shao et al., 2018). In Chinese medicinal herbs, BEA is the frequently detectable toxin with a 20% incidence (Hu and Rychlik, 2014). The contamination of BEA in Portuguese cereal-based foods was evaluated by Blesa et al. (2012). The percentage of BEA was 1.6%. For the total samples, the mean contamination of BEA was 0.1 mg/kg BEA. The wheat-based samples showed higher levels and greater prevalence than any other cereals monitored. The occurrence of BEA in analyzed pasta and multi cereal baby food samples from the Campania region (Italy) were below 68% (Juan et al., 2013a). A high incidence (70.3%) and high contamination levels (< 1100 μg/kg) of BEA in multi cereal baby food and its intakes could represent a risk for the infant population.

BEA (6.2–844 μg/kg) is one of the most predominant mycotoxins in food and diet (wheat and maize-based products) from Mediterranean area (Serrano et al., 2012). In the main markets of Abidjan, Bouake, and Korhogo, France, 91% of the rice, maize and peanut samples were contaminated with more than one mycotoxin including BEA (79% of the samples) (Manizan et al., 2018). The peanut paste samples represented the highest risk to consumer health followed by maize and rice samples. BEA (2 μg/kg) were detected in wheat ears randomly collected during year 2014 and 2015 from various localities in the Czech Republic (Sumikova et al., 2017). Besides these countries, BEA was also monitored in different Italian organic cereals and cereal products. Around 80% of analyzed samples contained with BEA (6.7–41 μg/kg) (Juan et al., 2013b).

The levels of BEA were analyzed in feed ingredients and compound feeds that were distributed throughout Korea during 2006 and 2007 (Lee et al., 2010). Twenty seven percentage of feed ingredients were contaminated with BEA at levels of 0.01–1.80 μg/g. The mean concentration of BEA was highest in bran feeds (0.76 μg/g). In compound feeds, 33% of samples were contaminated with BEA at levels of 0.01–4.66 μg/g. The mean BEA concentration was higher in swine (0.74 μg/g) and dairy cattle (0.72 μg/g) feeds than in beef cattle (0.43 μg/g) and chicken (0.37 μg/g) feeds. BEA is also detected in Irish farm silages (21.8 μg/kg) (McElhinney et al., 2015) and corn-dried distiller's grains in Thailand (350 μg/kg) (Tansakul et al., 2013).

Currently, the occurrence data for BEA is still limited and only available from certain parts of the world. Quantitative skin permeability data showed that BEA penetrates through the human skin and cross blood brain barrier to exert toxic effects on human (Taevernier et al., 2016b). However, as an emerging toxin, the toxicokinetic data and the risk assessment of BEA on humans are rarely reported (Bertero et al., 2018). Therefore, it is still difficult to conduct a full risk assessment for BEA. The BEA occurrence in grains and grain-based foodstuffs in recent years are summarized in Table 1.

**Table 1:** Beauvericin occurrence in grains and grain-based foodstuffs from different countries.

**Commodity**	**Incidence (%)**	**Concentrations (μg/kg)**	**Country**	**References**
Wheat	36	7.1 (max 144.8)	Belgium	Decleer et al., 2018
Wheat	82	5.2 (max 13.5)	Belgium	Decleer et al., 2018
Wheat	2	0.07	Romania	Stanciu et al., 2017
Wheat flour	1	0.3	Japan	Yoshinari et al., 2016
Corn grits	34.1	3.8	Japan	Yoshinari et al., 2016
Oats	73	31 (max 110)	Denmark	Svingen et al., 2017
Barley	7	10 (max 130)	Denmark	Svingen et al., 2017
Durum wheat	87	3.8 (max 56.4)	Italy	Covarelli et al., 2015
Soft wheat	100	26.8 (max 52.8)	Italy	Covarelli et al., 2015
Wheat-based baby foods	9.09	1.18 (max 21.3)	Italy	Juan et al., 2014
Multicereal baby foods	17	5.7	Italy	Juan et al., 2013a
Wheat	26.32	12.8 (max 35)	Italy	Juan et al., 2013b
Rye	45.45	2.72 (max 16.5)	Italy	Juan et al., 2013b
Ginger	20	19	China	Hu and Rychlik, 2014
Feed	27	0.48 (max 1.8)	Korea	Lee et al., 2010
Rice	3	54.7	Mediterranean region (Italy, Morocco, Tunisia, Spain)	Serrano et al., 2012
Wheat based products	18.46	Max 844	Mediterranean region	Serrano et al., 2012
Maize	36.36	Max 8,200	Slovakia	Srobarova et al., 2002
Whole-grain dry pasta	10	10.14	Spain	Serrano et al., 2013
Wheat	42.9	0.17–3.5	Spain	Meca et al., 2010
Maize	19.4	0.17–59	Spain	Meca et al., 2010
Cereal	1.6	0.1	Portugal	Blesa et al., 2012
Rice	75.5	3.8–26.3 mg/kg	Morocco	Sifou et al., 2011
Rice	4.28	210–19,600	Morocco	Sifou et al., 2011
Wheat	3.22	Max 2,000	Morocco	Zinedine et al., 2011
Wheat	61.62	4.1 (max 68.8)	The Netherlands	Van der Fels-Klerx, 2009
Barley	7.14	19 (max 19)	Finland	Jestoi et al., 2004
Oats		10 (max 220)	Finland	Uhlig et al., 2007
Winter wheat	77.41	3.2 (max 13.0)	Sweden	Lindblad et al., 2013
Oats		19 (max 120)	Norway	Uhlig et al., 2006
Maize	75	Max 45	USA	Wu and Smith, 2007
Maize	75	Max 40	South Africa	Sewram et al., 1999
Maize	9.10	10–1,864	Croatia	Jurjevic et al., 2002
Maize	100	1,800–3,6890	Poland	Kostecki et al., 1995
Maize	75	Max 45	USA	Wu and Smith, 2007

## Bioactivity

BEA shows cytotoxic, apoptotic, anticancer, anti-inflammatory, antimicrobial, insecticidal, and nematicidal activities. BEA is also an ionophoric cyclodepsipeptide which forms complexes with cations and increases the permeability of biological membranes (Massini and Näf, 1980; Toman et al., 2011; Wätjen et al., 2014; Lu et al., 2016). Because BEA has very efficient effects in the anticancer, antimicrobial, and insecticidal activities, this mycotoxin is considered to have the potential to be developed as a medicine or a pesticide. The various kinds of bioactivities of BEA are due to some unique active mechanisms, including the ions transport, oxidative stress, and autophagy (Wang and Xu, 2012).

### Cytotoxic activity

The cytotoxic effects of BEA have been studied by several authors. BEA (10 μM) can induce significant toxicity in TM-Luc 102 and Caco-2 cells (Fernández-Blanco et al., 2016). BEA shows toxicity in oocytes and cumulus cells at concentrations exceeding 0.5 μM (Schoevers et al., 2016). BEA significantly inhibits bovine granulosa cell proliferation at 3, 6, and 10 μM (Albonico et al., 2017). At 30 μM, BEA shows inhibitory effects on IGF1-induced CYP1 and CYP1 mRNA abundance (Albonico et al., 2017).

The mechanism of the BEA cytotoxicity is not fully understood. Researchers showed that BEA reduces cell viability correlating with the ROS generation and malondialdehyde formation (Ferrer et al., 2009). Similarly, in the study of Jow et al. (2004), BEA induces human leukemia cell death and this process has underwent an apoptotic pathway. In their study, CCRF-CEM cells were treated with BEA (1–10 μM) for 24 h, BEA-induced cell death exhibited a dose and time-dependent manner. This incidence of nuclear fragmentation and apoptotic body formation were significantly increased. Cytosolic caspase-3 activity and the release of Cyt c from mitochondria were also observed. The cellular toxicity targets of BEA are the mitochondrion and the homeostasis of potassium ions (Tonshin et al., 2010). In isolated rat liver mitochondria, exposure to BEA depleted the mitochondrial transmembrane potential, uncoupled oxidative phosphorylation, induced mitochondrial swelling and decreased Ca^2+^ retention capacity of the mitochondria (Tonshin et al., 2010). BEA can alter the mitochondrial membrane potential and produces DNA strand breakage (Mallebrera et al., 2016). Moreover, Mallebrera et al. (2016) showed that, BEA exposure for 24 h arrested the G_0_/G_1_ phase of CHO-K1 cell cycle and produces apoptosis. It seems that BEA-induced apoptosis is controlled by a balanced expression between apoptotic (Bax, Bad) and antipoptotic (Bcl-2) proteins (Mallebrera et al., 2018).

Importantly, a pre-treatment of the Ca^2+^ chelator can significantly increase the survival rate of the cells. Thus, intracellular Ca^2+^ plays an important function, perhaps as a mediator in the induced cell death signaling (Jow et al., 2004). A very recent study (Manyes et al., 2018) further showed that the cytotoxicity of BEA involves mitochondrial alterations, apoptosis, and cell cycle disturbances, since they observed a much higher percentage of apoptotic rate. Caspase-3 and 7 were highly activated and BEA arrested the S phase of the cell proliferation. Moreover, we further suspect that the BEA-induced cytoxicity has a relationship with autophagy, although the study of autophagy in BEA has rarely been reported. Since autophagy has a complex function in cell proliferation (Wu et al., 2018), therefore, it is quite interesting if some studies of autophagy in the cytotoxicity of BEA are conducted in the future.

On the other hand, BEA can increase ion permeability in biological membranes by forming a complex with essential cations (Ca^2+^, Na^+^, K^+^), which may affect the ionic homeostasis (Jow et al., 2004). Zhan et al. (2007) further showed that BEA is capable of inhibiting the metastatic prostate cancer and breast cancer cells and has antiangiogenic activity in HUVEC-2 cells.

The results of the studies suggest that BEA could be neurotoxic, but almost nothing is known about the mechanism of its neurotoxic effect (Zuzek et al., 2016). In the paper of Zuzek et al. (2016), BEA was shown to disrupt neurotransmission at the motor endplate. BEA reduced the release of acetylcholine from the presynaptic terminal. BEA formed cation-selective channels thus could depolarize and inactivate voltage-dependent sodium Nav1.4 type channels (Kouri et al., 2003).

### Anticancer activity

Currently, the anticancer potential of BEA is a hot topic (Zhan et al., 2007; Lu et al., 2016). BEA inhibits migration of the metastatic prostate cancer ((PC-3M)), breast cancer (MDA-MB-231) cells and exhibits antiangiogenic activity in HUVEC-2 cells (Zhan et al., 2007). BEA was also reported to induce the apoptosis of human non-small cell lung cancer (NSCLC) A549 cells (Lin et al., 2005), KB, and KBv200 cells (Tao et al., 2015). The anticancer activities of BEA are mainly studied by the working group of Jow [see Jow et al., 2004; Lin et al., 2005; Tang et al., 2005; Chen et al., 2006)]. BEA induced apoptosis through mitochondrial pathways, including decrease of ROS generation, loss of mitochondrial membrane potential, release of cytochrome c, and activation of Caspase-9 and−3 (Tao et al., 2015).

**Figure 4:**
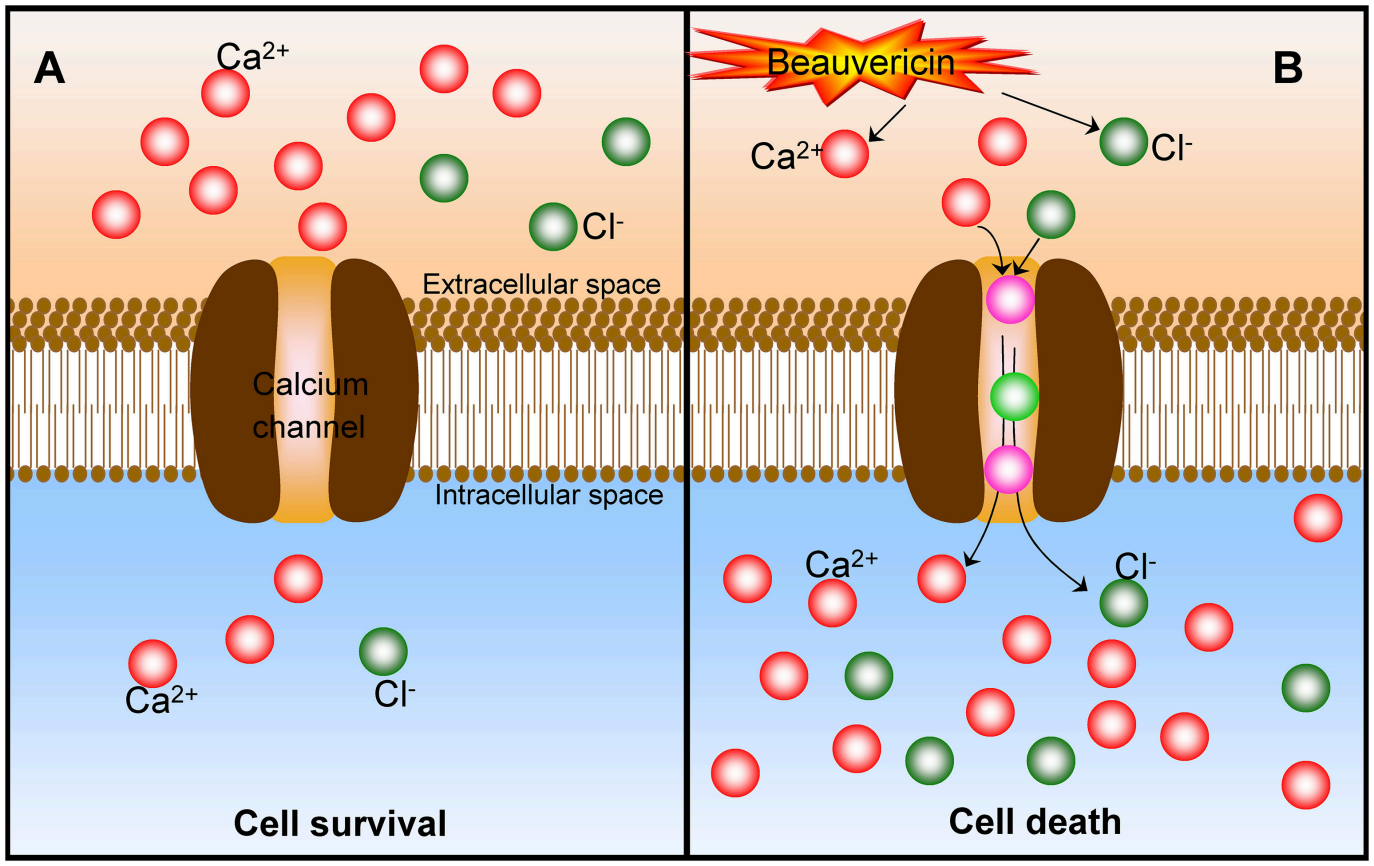
The mechanism of Ca^2+^ in the beauvericin-induced cancer cell death. **(A)** At normal status, there is a much higher extracellular Ca^2+^ content, and cancer cells keep surviving. **(B)** After the exposure of beauvericin, the extracellular Ca^2+^ are motivated to transfer into the intracellular through the calcium channel and induce the cancer cell apoptosis and death. Beauvericin can activate Ca^2+^-activated Cl^−^ currents and induce cell death as well.

As discussed, the mechanism of BEA-induced cancer cell apoptosis involves multiple cellular/molecular pathways and pro- and anti-apoptotic Bcl-2 family proteins (Lu et al., 2016). Moreover, the BEA-induced cell death is mainly due to the increase of intracellular Ca^2+^ concentration (Lu et al., 2016). However, it should be further to identify whether the increase of intracellular Ca^2+^ (from extracellular or intracellular stores) is an important factor with the apoptotic pathway to conduct EA-induced cancer cell death. In a subsequent study (Chen et al., 2006), the effect of BEA on Ca^2+^ concentration [[Ca^2+^]i] and the underlying mechanisms responsible for the changes of [Ca^2+^]i in CCRF-CEM cells were further investigated. Indeed, BEA caused a rapid and sustained [Ca^2+^]i rise. Excess extracellular Ca^2+^ facilitated a BEA-induced [Ca^2+^]i rise in the bathing medium. It is noteworthy that the voltage-dependent Ca^2+^ channel blocker or intracellular Ca^2+^ depletion does not affect the BEA-induced [Ca^2+^]i rise. Thus, BEA should act as a potent Ca^2+^ mobilizer by stimulating an extracellular Ca^2+^ influx and inducing the cancer cell apoptosis (Figure 4). Similarly, BEA can activate Ca^2+^-activated Cl^−^ currents and induces cell death in *Xenopus* oocytes via the influx of extracellular Ca^2+^ (Tang et al., 2005).

As mentioned, BEA induces apoptosis in a variety of cancer cell lines, but the underlying mechanism(s) is still not fully understood. Lin et al. (2005) showed that BEA-induced human NSCLC A549 cell apoptosis through the up-regulation of cytokines Bax and p-Bad and down-regulation of p-Bcl-2. Also, the reduction of mitochondrial membrane potential, the activation of caspase 3 and cytochrome c release were observed and should be involved in the mechanism of BEA-induced cancer cell death (Jow et al., 2004; Lin et al., 2005). However, in the study of Tao et al. (2015), the decrease of protein level Bcl-2 and Bax after treatment with BEA (3–12 μM) for 48 h was not observed in KB cells and KBv200 cells. This different effect on pro and anti-apoptotic Bcl-2 family proteins induced by BEA might be relevant to various characteristics of the cell lines.

Oxidative stress is one important effect involved in BEA anticancer activity (Prosperini et al., 2013). BEA can increase the ROS level at an early stage. BEA induced cancer cell death via an apoptotic process with reduced G0/G1 phase and with an arrest in G2/M. Moreover, BEA caused DNA damage after 12.0 μM concentration. Also, BEA exposure produces DNA strand breakage and induces CHO-K1 cell apoptosis (Mallebrera et al., 2016). In a very recent study (Escrivá et al., 2018), BEA was further shown to induce mitochondrial damage to affect the respiratory chain through the caspase cascade in Jurkat cells. However, on the contrary, one research group Dornetshuber et al. (2009) demonstrated that the oxidative stress and DNA interactions were not involved in BEA-mediated apoptosis. The reasons are not clear but are possibly due to the different cell lines, BEA concentrations, and functioning times used in their specific studies.

Some signaling pathways are involved in the anticancer mechanisms of BEA (Wätjen et al., 2014; Lu et al., 2016). For example, in cancer cell lines HepG2 and H4IIE, BEA reduced the ERK and NF-κB protein expression and promoted JNK phosphorylation (Wätjen et al., 2014). As known, deregulated NF-κB activity contributes to many human diseases, including tumors. Since NF-κB is a transcription factor which is responsible for cell survival, this inhibition may also contribute to the toxic effects of BEA. BEA induces an increased JNK phosphorylation, which is generally associated with cell death. Additionally, numerous protein kinase in the signaling transduction pathway showed a selective inhibition of Src kinase by BEA (Wätjen et al., 2014). MEK1/2-ERK42/44-90RSK pathway plays an important role in the mechanism of BEA-induced NSCLC A549 cancer cell apoptosis (Lu et al., 2016). Further, BEA can decrease the ERK and NF-κB phosphorylation but increase JNK phosphorylation in H4IIE cells. During a screening of 21 protein kinases, BEA shows selective inhibition of Src kinase in signal transduction pathways (IC_50_ = 9.8 μg/ml) (Wätjen et al., 2014). Recently, Lu et al. (2016) reported that BEA induces NSCLC A549 cancer cell apoptosis through the mitogen-activated protein kinase pathway (MAPK), BEA can also activate MEK1/2-ERK42/44-90RSK crosstalk signaling pathway which can induce A549 cell cycle arrest in the S phase and apoptosis (Lu et al., 2016). *In vivo*, BEA reduces the levels of TNF-α and IFN-γ in mice serum. BEA suppresses IFN-γ-STAT1-T-bet signaling and leads to T cell apoptosis (Massini and Näf, 1980). Compared with other mycotxins, for example, trichothecenes (Wu et al., 2017), the study of the signaling pathway of BEA in anicancer mechanisms is relatively limited. More studies on the signaling pathways in the BEA cytotoxicity are warranted.

It should be noted that most of the above studies are performed *in vitro* using cancer cell lines and the *in vivo* studies are urgently needed to identify the anticancer capacity of BEA. Recently, Heilos et al. (2017) have tested the *in vivo* anticancer effects of BEA by treating mice bearing murine CT-26 or human KB-3-1-grafted tumors, respectively. Decreased tumor volumes and weights in BEA-treated mice without any adverse effects were observed. BEA accumulation was also detected in tumor tissues. Moreover, a significant increase of necrotic areas within whole tumor sections of BEA-treated mice was observed, confirming its promising role as a novel natural compound for anticancer therapy.

Thus, BEA shows promising anticancer potential through investigation of different cancer cell lines. Normally, BEA induces extracellular Ca^2+^ translocated into the cytosol, which leads to an increase intracellular Ca^2+^ level. The increased Ca^2+^ may activate a series of signaling pathways, for example, MAPK, NF-κB, and decreases the mitochondrial transmembrane potential, release of Cyt c, and activates caspase, therefore further promotes the cancer cell apoptosis. However, up to date, we have rarely seen the *in vivo* data of anticancer of BEA. The *in vivo* data is very important since we need to know that whether BEA is stable and active in the body. Moreover, the toxicity of this product is an important issue for consider. Thus, the information of the *in vivo* anticancer activity of BEA are urgently needed. In addition, in the future, the study of the BEA-monoclonal antibodies (mAb) should be a promising strategy in anticancer therapies.

### Anti-inflammatory activity

**Figure 5:**
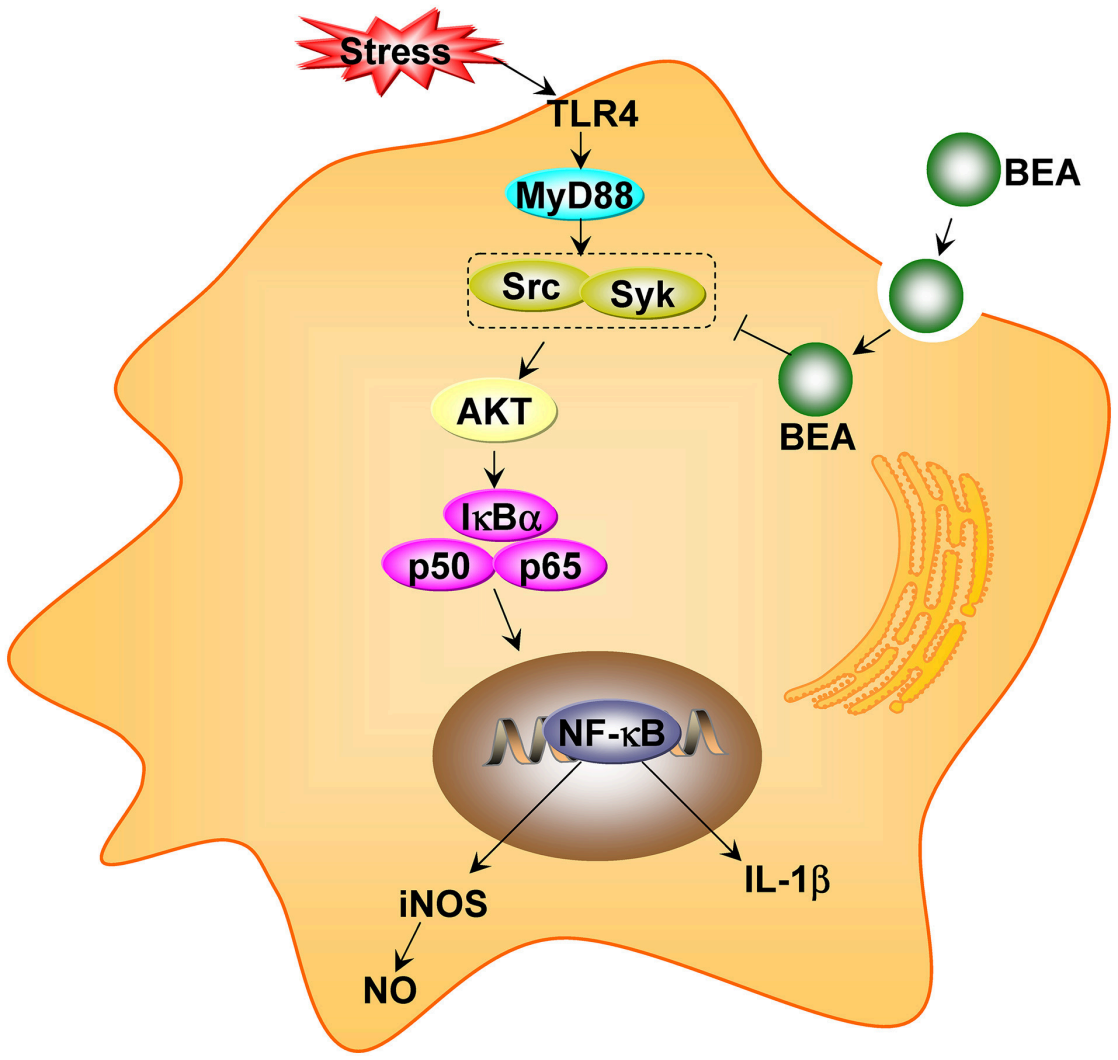
The proposed anti-inflammatory pathway of beauvericin (BEA) in macrophages (adapted from Yoo et al., 2017).

BEA has anti-inflammatory activities and it inhibits inflammatory responses by inhibiting the NF-κB pathway. In a recent study by Yoo et al. (2017), BEA blocked the production of nitric oxide (NO) in lipopolysaccharide-treated RAW264.7 cells without inducing cell cytotoxicity. Moreover, BEA inhibited the nuclear translocation of the NF-κB subunits p65 and p50. Luciferase reporter gene assays demonstrated that BEA suppressed MyD88-dependent NF-κB activation. By analyzing upstream signaling events for NF-κB activation, overexpression of Src and Syk were observed and these two enzymes were the potential targets of BEA. Thus, BEA is a strong anti-inflammatory agent that attenuates NF-κB-dependent inflammatory responses by suppressing enzymes Src and Syk (Figure 5) (Yoo et al., 2017).

The current therapeutic goals of colitis are aimed at reducing the occurrence of the main symptoms and preventing further development of the disease. BEA has a therapeutic role in the colitis. Inflammatory bowel disease is usually accompanied by abnormal secretion of inflammatory cytokines. These abnormally secreted cytokines also promote inflammation and play an important role in the occurrence and development of inflammatory bowel disease. BEA can inhibit the expression of L-12, IL-1β, and IFN-γ in enteritis colon tissue with enteritis (Wu et al., 2013). Because of the anti-inflammatory effect, BEA shows a promising capacity for the treatment of colonic inflammation by targeting the PI3K/Akt pathway (Wu et al., 2013). BEA decreased serum levels of TNF-α and interferon IFN-γ and suppressed T-cell proliferation. BEA further reduced the activation of IFN-γ-STAT1-T-bet signaling and led to T cell apoptosis by suppressing Bcl-2 and increasing cleavage of caspases and PARP (Wu et al., 2013). Therefore, targeting PI3K/Akt in the activated T cells by BEA maybe a novel therapy for Crohn's disease.

### Antimicrobial activity

BEA has a strong antibacterial activity against Gram-positive and Gram-negative pathogenic bacteria (Nilanonta et al., 2000, 2002; Meca et al., 2010). Also, numerous bacterial strains without distinction between Gram-positive and Gram-negative bacteria, are inhibited by BEA as well, for example, *Bacillus* spp., *Bifidobacterium adolescentis, Clostridium perfringens, Paenibacillus* spp., and *Peptostreptococcus* spp. (Castlebury et al., 1999; Xu et al., 2010). BEA exhibits minimum inhibitory concentration (MIC) values of 0.8–1.6 mg/mL against *M. tuberculosis*, and IC_50_ values of 1.3–2.4 mg/mL against *P. falciparum* (Nilanonta et al., 2002). BEA showed remarkable activity against two Gram-negative strains (*B. cereus* and *S. typhimurium*) with respective MIC values of 3.12 and 6.25 μg/ml (Dzoyema et al., 2017). As described by Xu et al. (2010), the median effective inhibitory concentration values of BEA against 6 test bacteria (*B. subtilis, S. haemolyticus, P. lachrymans, A. tumefaciens, E. coli*, and *X. vesicatoria*) were between 18.4 and 70.7 μg/mL. In another study (Zhang et al., 2016), BEA showed an inhibitory effect on three human pathogenic microbes, *C. albicans, E. coli*, and *S. aureus*. In particular, BEA exhibited the strongest antimicrobial activity against *S. aureus* with MIC values of 3.91 μM.

Regarding its antibacterial mechanism of BEA, the antibacterial effect of BEA is different from other antibiotics. Unlike most antibiotics, the bacterial cell wall is not the antibacterial mode of BEA activation. Cell organelles or enzyme systems are the targets of BEA (Prince et al., 1974; Wang and Xu, 2012). Based on the antibacterial activity against plant pathogens (Xu et al., 2010), BEA could be utilized in the control of non-food crop diseases and to solve the problems of drug resistance (Tong et al., 2016).

BEA also has a very effective antifungal activity. An administration of BEA with ketoconazole shows an antifungal effect with more than 100-fold higher than that by a single application (Zhang et al., 2007). The mechanism of antifungal activity of BEA has been studies by numerous studies (Mei et al., 2009; Shekhar-Guturja et al., 2016a; Tong et al., 2016). The synergetic effect is not due to their pharmacokinetic interactions (Mei et al., 2009). Tong et al. (2016) further reported that BEA can counteract multidrug resistant *Candida albicans* by blocking ABC transporters. As a drug efflux pump modulator, BEA reverses the multi-drug resistant phenotype of *C. albicans* by blocking the ATP-binding cassette transporters. BEA shows fungicidal activity by elevating intracellular Ca^2+^ and ROS (Tong et al., 2016). Recently, a powerful strategy to enhance antifungal efficacy against human fungal pathogens was investigated by using BEA (Shekhar-Guturja et al., 2016a,b). BEA potentiated the activity of azole antifungals against azole-resistant *Candida* isolates *via* blocking multidrug efflux and inhibition of global regulator TORC1 kinase; thereby activating protein kinase CK2 and inhibiting the molecular chaperone Hsp90. Pdr5 substitutions enable BEA efflux (Shekhar-Guturja et al., 2016a). BEA itself was effluxed *via* Yor1. Zcf29 bound to and regulated the expression of multidrug transporter genes (Shekhar-Guturja et al., 2016b). Beyond drug resistance, BEA blocked the *C. albicans* morphogenetic transition from yeast to filamentous growth in response to diverse cues. BEA repressed the expression of many filament-specific genes, including the transcription factor BRG1 (Shekhar-Guturja et al., 2016b). Thus, BEA simultaneously targets drug resistance and morphogenesis provides a promising strategy to combat life-threatening fungal infections. The antifungal activity of BEA alone is very weak but can be greatly increased in combination with ketoconazole or miconazole (Fukuda et al., 2004; Zhang et al., 2007). The structure-activity relationship (SAR) of BEA should be further studied to explore its antimicrobial activity.

### Insecticidal and nematicidal activity

BEA shows a very promising insecticidal potential. In 1969, the working group of Hamill et al., firstly discovered the insecticidal activity of BEA against a model organism *Artina salina* (Hamill et al., 1969). Similarly, other authors investigated the insecticidal effect of BEA on *Aedes aegypti* (Grove and Pople, 1980), *Calliphora erythrocephala* (Daniel et al., 2017), *Spodoptera frugiperda* (Fornelli et al., 2004), and *Lygus* spp. (Leland et al., 2005). Although BEA was claimed to have insecticidal properties (Hamill et al., 1969), it is a pity that little detailed information has been published. Currently, BEA was confirmed against model organism to study insecticidal activity, *Artemia salina* (Hamill et al., 1969), against *C. erythrocephala* (Grove and Pople, 1980), *A. aegypti* (Wang and Xu, 2012), *Lygus* spp. (Leland et al., 2005), *S. frugiperda* (Fornelli et al., 2004), and *Schizaphis graminum* (Ganessi et al., 2002).

Up to date, there are very few reports about the insecticidal mechanism of BEA. Although BEA has similar chemical structures with other cyclic hexadepsipeptide mycotoxins, this mycotoxin is more effective against *A. aegypti* and suggesting a unique mechanism of action exists (Grove and Pople, 1980). The methanolic and ethyl acetate-methanolic extracts of *B. bassiana* showed larvicidal activity against 3rd instar of *A. aegypti* (Daniel et al., 2017). Cyclodepsipeptides are the active principles for the larvicidal action. BEA is a potential insecticidal component in the formulations for the Dengue and Zika vector.

BEA shows a promising nematicidal activity. Culture filtrates of *B. bassiana* were evaluated for nematicidal activity against the northern root-knot nematode (*Meloidogyne hapla*) (Liu et al., 2008). The nematode population densities and subsequent gall formation and egg-mass production by *M. hapla* were thoroughly decreased by the filtrates (Liu et al., 2008). Zhao et al. (2013) further demonstrated that the culture filtrate of different isolates of *B. bassiana* had different levels of activities against the same nematode, and the same culture filtrate had selective toxicity against different nematodes (Kepenekci et al., 2017). In addition, BEA shows nematicidal activities against the pine wood nematode *B. xylophilus* and the free-living nematode *C. elegans* (Shimada et al., 2010). Very recently, a new-to-nature octa-BEA showed up to very effective antiparasitic activity against *Leishmania donovani* and *Trypanosoma cruzi* (Steiniger et al., 2017). However, its antiparasitic mechanism is poorly understood and needs more investigations.

## Concluding remarks

BEA as a mycotoxin has different kinds of biological activities. It has a therapeutic potential for cancer, and viral or bacterial infections, as well as, other deadly diseases. Regarding these appealing pharmacological properties, BEA and its metabolites fulfill the requirements to be considered for further pre-clinical development as the treatment option for cancers. However, this mycotoxin is a common part of food and food ingredients as the contaminant, and there is still no clear answer of the question on whether long-term consumption of the low-dose of mycotoxin BEA can cause harm to humans and animal health. Detailed studies on the consequences of chronic and bolus BEA exposure are eagerly needed. Furthermore, occurrence data for BEA is still quite limited and only available from certain parts of the world. Therefore, currently, it is not possible to carry out a full risk assessment for BEA. In the future, it is necessary to evaluate the effectiveness of BEA as a substance that can be used in agriculture or medicine. The investigation on the BEA-mAb in anticancer therapies should be encouraged.

## Author contributions

QW, EN, and JP wrote the manuscript. KK reviewed and edited the manuscript. All the authors critically reviewed the literature and contributed to drafting the manuscript.

### Conflict of interest statement

The authors declare that the research was conducted in the absence of any commercial or financial relationships that could be construed as a potential conflict of interest.
